# Drought responsiveness in six wheat genotypes: identification of stress resistance indicators

**DOI:** 10.3389/fpls.2023.1232583

**Published:** 2023-09-13

**Authors:** Asma Guizani, Hend Askri, Maria Laura Amenta, Roberto Defez, Elyes Babay, Carmen Bianco, Nicoletta Rapaná, Mariella Finetti-Sialer, Fatma Gharbi

**Affiliations:** ^1^ Laboratory of Mycology, Pathologies and Biomarkers LR16ES05, Faculty of Sciences of Tunis, University of Tunis El Manar, Tunis, Tunisia; ^2^ Laboratory of Valorization of Non-Conventional Water (LR16INRGREF02), National Institute of Rural Engineering, Water and Forestry, Carthage University, Tunis, Tunisia; ^3^ Institute of Biosciences and BioResources, National Research Council, Naples, Italy; ^4^ Laboratory of Cereals and Food Legumes, National Gene Bank of Tunisia (BNG), Tunis, Tunisia; ^5^ Agricultural Applied Biotechnology Laboratory (LR16INRAT06), Institut National de la Recherche Agronomique de Tunisie (INRAT), University of Carthage, Tunis, Tunisia; ^6^ Institute of Biosciences and BioResources, National Research Council, Bari, Italy

**Keywords:** cell wall integrity, drought, osmotic adjustment, stomatal regulation, transcriptional analysis, water relations, wheat

## Abstract

**Introduction:**

Wheat (*Triticum aestivum* L.) is among the world’s most important staple food crops. In the current climate change scenario, a better understanding of wheat response mechanisms to water stress could help to enhance its productivity in arid ecosystems.

**Methods:**

In this study, water relations, gas exchange, membrane integrity, agronomic traits and molecular analysis were evaluated in six wheat genotypes (D117, Syndiouk, Tunisian durum7 (Td7), Utique, Mahmoudi AG3 and BT) subjected to drought-stress.

**Results and discussion:**

For all the studied genotypes, drought stress altered leaf area, chlorophyll content, stomatal density, photosynthetic rate and water-use efficiency, while the relative water content at turgor loss point (RWC0) remained stable. Changes in osmotic potential at turgor loss point (Ψπ^0^), bulk modulus of elasticity (Ɛmax) and stomatal regulation, differed greatly among the studied genotypes. For the drought-sensitive genotypes AG3 and BT, no significant changes were observed in Ψπ^0^, whereas the stomatal conductance (gs) and transpiration rate (E) decreased under stress conditions. These two varieties avoided turgor loss during drought treatment through an accurate stomatal control, resulting in a significant reduction in yield components. On the contrary, for Syndiouk, D117, Td7 and Utique genotypes, a solute accumulation and an increase in cell wall rigidity were the main mechanisms developed during drought stress. These mechanisms were efficient in enhancing soil water uptake, limiting leaf water loss and protecting cells membranes against leakage induced by oxidative damages. Furthermore, leaf soluble sugars accumulation was the major component of osmotic adjustment in drought-stressed wheat plants. The transcriptional analysis of genes involved in the final step of the ABA biosynthesis (AAO) and in the synthesis of an aquaporin (PIP2:1) revealed distinct responses to drought stress among the selected genotypes. In the resistant genotypes, PIP2:1 was significantly upregulated whereas in the sensitive ones, its expression showed only a slight induction. Conversely, the sensitive genotypes exhibited higher levels of AAO gene expression compared to the resistant genotypes. Our results suggest that drought tolerance in wheat is regulated by the interaction between the dynamics of leaf water status and stomatal behavior. Based on our findings, Syndiouk, D117, Utique and Td7, could be used in breeding programs for developing high-yielding and drought-tolerant wheat varieties.

## 1 Introduction

In the context of global climate change, more frequent and severe drought episodes are expected in the coming years (Wood et al., 2023). These events will negatively affect crop growth and therefore food production. Over 20% of the world’s agricultural areas suffer from drought problems (Mansoor et al., 2022), and Tunisia is one of the countries with the lowest availability of water in the world (Hachani et al., 2022). Since many rivers and dams have already dried up, Tunisia is using up to 80% of the available water resources in agriculture (Douh et al., 2022). Wheat is consumed as a fundamental food grain all over the world (Hussein et al., 2023), with Tunisia being an important consumer. Thus, the country is facing a great challenge to preserve national food security under serious drought threats. Plant responses in arid conditions involve complex functional and structural adaptations. For wheat crops, different levels of drought-tolerance mechanisms, involving changes in physiological processes of plant leaves, have been observed. Metabolic disruptions and great yield loss result from the activation of different mechanisms, including water and minerals uptake, CO_2_ assimilation, transpiration, stomatal conductance, chlorophyll concentrations, stomatal density and cell wall integrity (Hachani et al., 2022; Srinatha et al., 2023). Various study have shown differences in the capacity of plant species to perform stomatal regulation, osmotic adjustment and to change the leaf tissues elastic properties (Martínez et al., 2007; Hessini et al., 2008; Sampaio Filho et al., 2018; Leuschner et al., 2019; Wood et al., 2023). The determination of water relations and leaf gas exchange parameters are fundamental to analyze and quantify the effects of drought on the cell and tissue physiology of wheat plants and to understand how they impact on their survival, growth, and productivity. Leaf turgor loss point (Ψ_π_^0^) was identified as a key parameter to evaluate the response of many plants to drought stress (Wood et al., 2023). During water deficits, isohydric species maintained a constant Ψ_π_^0^ through sensitive stomatal regulation, reduced stomatal conductance (gs) and transpiration, and was more depended on stored carbohydrates to meet continued carbon demands. On the other hand, anisohydric plants showed marked drops in Ψ_π_^0^, high gs levels, and increased evaporative demand, as stomata close later and reached more negative xylem water potentials. Plants often regulate the use of water by balancing water supply and demand to safely maintain leaf osmotic potential above Ψ_π_^0^ (Wood et al., 2023). As solutes accumulate, the turgor pressure increases until the cell reaches Ψ_π_ equilibrium in the immediate surroundings (Juenger and Verslues, 2023). This effect can derive from either simple passive solute concentration resulting from dehydration, or from net solute accumulation, considered as active osmotic adjustment (OA) (Patakas et al., 2002). The compounds involved in OA differ widely in plants species and appear to be organic within wheat plants (Hussein et al., 2023). Besides differences in stomatal regulation, anisohydric and isohydric plants differ in the elastic properties of leaf tissues. Cell wall elasticity is considered one of the most important physiological mechanisms of adaptation to water stress (Martínez et al., 2007). At the cellular level, plants with lower Ψ_π_^0^ tended to cope with drought stress through changes in the elasticity of cell walls to maintain turgor (Leuschner et al., 2019). Rigid cell walls help maintaining lower water potential at any given volume compared to elastic ones. This effect can lead to increased water potential gradient between the soil and the plant, thereby promoting more effective water uptake from drying soils and/or accelerating recovery after re-watering (Patakas et al., 2002; Hessini et al., 2008).

The physiological mechanisms underlying stomatal response most likely involve the accumulation of the drought hormone abscisic acid (ABA) (Munemasa et al., 2015; Ali et al., 2020; Aslam et al., 2022). Recent studies on the isohydric specie *Arabidopsis thaliana* have shown that ABA reduces leaf hydraulic conductance through the downregulation of aquaporin activity (Shatil-Cohen et al., 2011; Coupel-Ledru et al., 2017). These results suggest that ABA promotes stomatal closure either through a local biochemical mechanism on the guard cells, or via a remote hydraulic impact with a decrease in water permeability within the bundle sheath. It has been hypothesized that the hydraulic effect of ABA could underlie the apparent interaction between hydraulics and ABA on the stomatal control of isohydric species and therefore could give rise to the genetic differences between isohydric and anisohydric behaviors. It has also been reported that the molecular mechanism determining the conversion of plants from isohydric behavior to a more tolerant anisohydric one may involve the expression of aquaporins (AQPs) (Sade et al., 2009). AQPs are considered as the main channels for the transport of water, as well as small neutral solutes and CO_2_, through the plant cell membrane (He et al., 2023).

A better comprehension of the morphoanatomical and physiological basis of drought stress in wheat crops could facilitate the selection or creation of new germplasm resources to increase productivity in drought- prone areas. This study presents the findings of a comparative study on drought responses strategies of six wheat genotypes and highlighted the features that can be used for the selection of genotypes showing a water stress resistance. The objectives were: (i) to analyze water relation parameters obtained from pressure-volume (P-V) curves of plants subjected to optimal watering and drought conditions; (ii) to identify the compounds involved in leaf osmotic adjustment; (iii) to characterize the physiological processes and response strategies of the six wheat genotypes under drought stress conditions; and iv) to evaluate the expression level of key genes involved in drought stress response.

## 2 Materials and methods

### 2.1 Plant material and drought treatment

Seeds of four durum wheat (*Triticum turgidum* L. subsp*. durum*) genotypes (D117, Syndiouk, Tunisian durum7 and Mahmoudi AG3) and two bread wheat (*Triticum aestivum* L.) genotypes (Utique and BT) were selected. The genotypes selected in this study showed the most contrasting behavior in response to drought stress. Seeds were sterilized with a 20% sodium hypochlorite solution. They were sown in 1-L plastic pots (one seed per pot) containing a mixture of sand and peat (2:1 v/v). Plants were irrigated to 100% field capacity every two days by weight adjustment. Plants were grown under a transparent rainout shelter from January to June 2021. During the emergence-tillering phase, the peat provided the essential nutrients for the development of plants. During the growing season, the solar radiation gradually increased in the range 9−23 MJ m² day^-1^. The seeds used in this study were produced by a single seed descent (SSD) method and supplied by the National Gene Bank of Tunisia (NGBT). The drought treatment was carried out according to a completely randomized block design with ten replicates. At four-leaf-stage, 21-day-old wheat plants were subjected to drought stress for three weeks by suspending irrigation.

At the end of the treatment (6-week-old plants), all measurements were made on the youngest fully expanded leaves. Leaf SPAD and gas exchange measurements were performed using a non-destructive approach (n=5). Fresh leaves were used for pressure-volume curve analysis (n=3), estimation of electrolyte leakage (n=5), and stomatal imprint (n=5). Biochemical analyses, including the measurement of proline, soluble sugars, inorganic ions, and MDA contents (n=5), were carried out on leaves dried at 60°C for 72 hours. Soil moisture was restored until 100% field capacity with a half-strength Hoagland’s nutrient solution (Hoagland and Arnon, 1950) until spike harvest.

### 2.2 Environmental parameters

The maximum and minimum daily air temperature (T_max_ and T_min_), and relative humidity were obtained from the automatic weather station of the National Institute of Meteorology of Tunis-Carthage, which is the closest station to the experimental site at the Faculty of Science of Tunis. The data covers the entire growing season from January to June 2021. The average monthly temperatures ranged from 13 to 27°C, while the average relative humidity ranged from 53% to 70%. The cumulative GDD index, used to predict crop maturity, reached 2615°C throughout the wheat growing season. It is calculated based on the number of days (n) with a mean daily temperature higher than the base temperature (Tbase = 0°C), according to McMaster and Smika (1988) as follows:


$$\text{GDD}=\sum_{\text{n}1+\text{n}2\cdot \cdot \cdot \text{nn}}\frac{\left[T_{max}+T_{min}\right]}{2}-T_{base}$$

where *n* is the number of days when the mean daily temperature was higher than base temperature (T_base_ = 0°C).

### 2.3 Water relation parameters

At the end of the drought stress period, water relations parameters were determined from pressure-volume (P-V) curves using three randomly selected plants per treatment. This method is based on the evolution of the leaf cell water status under progressive dehydration. Fully expanded young leaves from all treatments were placed in distilled water and incubated at 25°C for 24h in the dark to determine the saturation weight (Wsat). The rehydration allowed the normalization of the relative water content of all samples. Measurements were carried out using simultaneous pressure chamber (SKPM 1400; Skye instruments Ltd., England, UK) and precision balance, according to the method described by Ritchie (1984). Nine pressure levels (starting from -0.2 MPa down to -4.0 MPa) were applied, and each level was maintained for 10 min. The sample were removed from the pressure chamber, weighed (W_f_) and placed in an oven at 60°C until a constant weight (W_dry_) was achieved. Thus, the pressure-volume curve was plotted: 1/Ψw = f(RWC) (Kubiske and Abrams, 1990).

RWCi were calculated using the following formula:


$$RWC_{i}=100-\left(\frac{C_{i}+(n_{i}\times E)}{W_{sat}-W_{dry}}\right)\times 100$$

Where, Ci is the cumulative weight of sap lost at pressure level i (g), and (ni×E) is the correction factor used to estimate the weight of the evaporated sap inside the chamber at level i.

**Figure 1:**
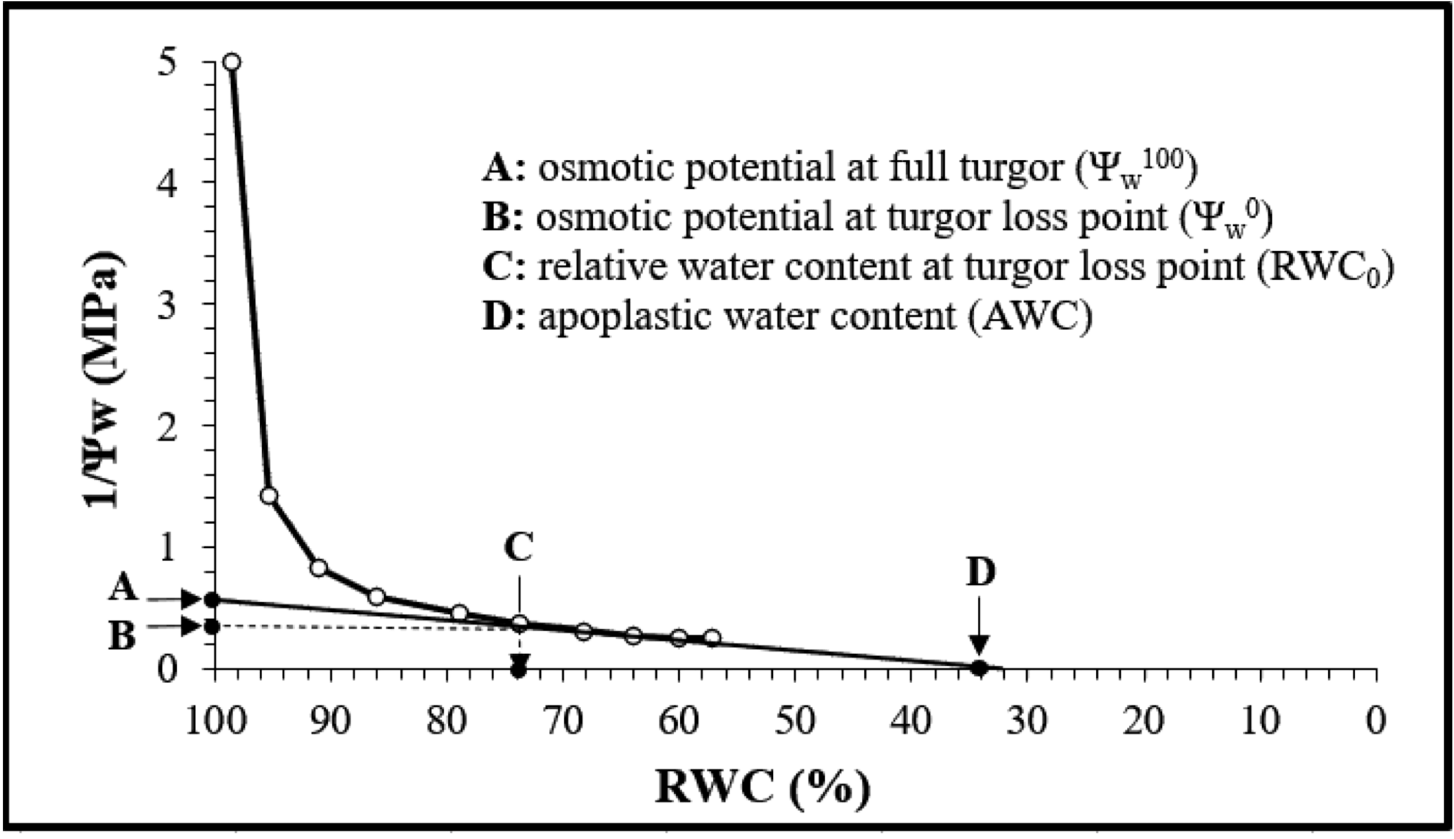
Pressure-volume curve generated from different measurements.

The generated P-V curves allowed the estimation of a set of variables as reported in **Figure 1** (Andersen et al., 1991).

The modulus of elasticity (Ɛmax) and the osmotic adjustment (OA) were calculated according to Hessini et al. (2008) and Martìnez et al. (2004) as follows:


$$\text{ϵmax}=\frac{\left({\text{ψ}}_{\text{π}}^{100}-{\text{ψ}}_{\text{π}}^{0})\times (1-\text{AWC}\right)}{\left(1-\text{RWC}0\right)}$$

$$\text{OA}={\text{Ψ}}_{\text{π}}^{100}(\text{control})-{\text{Ψ}}_{\text{π}}^{100}(\text{treated})$$

where Ψπ100 is the osmotic potential at full turgor, Ψπ0 is the osmotic potential at turgor loss point, RWC0 is the relative water content at turgor loss point, and AWC is the apoplastic water content.

### 2.4 Proline and soluble sugar contents in leaf tissues

The proline content was measured by a colorimetric method according to Bates et al. (1973), using L-proline as a standard. The level of total soluble carbohydrates was determined according to McCready et al. (1950) using a glucose solution (0.1g/L) as a standard.

### 2.5 Inorganic ion content

Inorganic ions (Na^+^, K^+^ and Ca^2+^) were extracted at room temperature by dissolving 25 mg of dry plant powder in 25 ml of 0.5% HNO_3_ (Soltani et al., 1992). After 72h, the mineral deposits were filtered. Na^+^ and K^+^ ions were assayed by flame emission photometry and Ca^2+^ content was determined using an atomic flame emission spectrophotometer.

### 2.6 Contribution of solutes to osmotic adjustment

The concentrations of soluble sugars, proline, and inorganic ions were calculated for symplastic water volume at full turgor as: SWC (%) = 100 -AWC where AWC was estimated by the pressure–volume technique. According to Patakas et al. (2002), we assumed that 40 µmol g^-1^ of symplastic water corresponds to 0.1 MPa. The contribution of each solute (OA_s_) to total osmotic adjustment (OA) was estimated using the formula:


$$\text{OA}(\%)=\left[\frac{([\text{S}]\text{stressed}-[\text{S}]\text{control}\times 0.1\times 100}{40}\right]/\text{OA}$$

Where [S] is the solute concentration (µmol g^-1^ of symplastic water).

### 2.7 Leaf gas exchange and water use efficiency

At the end of the drought treatment, net photosynthetic rate (A), stomatal conductance (gs), intercellular CO_2_ concentration (Ci), and transpiration rate (E) were determined for the youngest fully expanded leaf using LI-6400 portable photosynthesis system. Measurements were conducted during the timeframe of 10:00 am to 12:00 noon at the temperature of 26°C, the saturated light of 1044μmol m−2 s−1 (PPFD), and the atmospheric CO_2_ of 400 ppm.

Water use efficiency represents the unit of carbon gain for each unit of water lost, calculated as WUE = A/E (µmol CO_2_ mol⁻¹ H_2_O), while intrinsic water use efficiency is defined as the ratio of net CO_2_ assimilation to stomatal conductance and calculated as WUE_i_ = A/gs (µmol CO_2_ mol⁻¹ H_2_O).

### 2.8 Stomatal density and leaf chlorophyll content

After thoroughly cleaning the leaf surface, a thin layer of nail polish was applied. When the film was dried it was removed and mounted on a glass slide. Stomata per view were counted under a photomicroscope. Five view areas’ stomatal averages were computed. Number of stomata per mm^2^ was used to define the stomatal densities of the upper and lower leaf surfaces.

Total chlorophyll content was estimated through a portable chlorophyll meter (SPAD). Correlations between SPAD measurements and chlorophyll content (mg g⁻¹ FW) were assessed for a total of 12 measurements according to the following equation:


$$\text{y}=0.0042\text{x}-0.089({\text{R}}^{2}=0.9741).$$

Where x is the SPAD measurement and y is the chlorophyll content (mg g⁻¹ FW).

### 2.9 Lipid peroxidation and membrane permeability

Lipid peroxidation was estimated by quantifying leaf MDA content using thiobarbituric acid method reading the supernatant at 532 nm and 600 nm and using the extinction coefficient of 155 mM^-1^ cm^-1^ as described in Verma and Dubey (2003). To evaluate the membrane integrity by relative electrolyte leakage (EL), 100 mg of fresh samples were cut into segments and incubated for 24h in the dark and at room temperature in deionized water (control plants) or stressed plants exposed to 20% PEG 6000 (Bajji et al., 2002).

Electrical conductivity was measured 1h after incubation at 25°C (EC1), and at 100°C (EC2) by using Jenway 4510 Conductivity/TDS Meter. Leaf EL was expressed as the percentage of the total electrolyte content obtained after boiling the segments and calculated as according to the following equation: EL = (EC1/EC2) *100 (Lutts et al., 1996).

### 2.10 Agronomic traits

The number of grains per plant (GNP), the grain yield per plant (GY), and the weight of 1000 seeds were measured for the six wheat genotypes at the stage of maturity. Drought resistance indices were used as quantitative measurement to classify wheat varieties according to their drought tolerance. The biological yield was measured on plants dried in oven at 60°C for 72 h. Drought resistance indices were determined using the following equations:

(1) Tolerance $(\text{TOL})=Y_{p}-Y_{s}$ (Rosielle and Hamblin, 1981).
(2) Stress Susceptibility Index

$(\text{SSI})=\left[1-\left(\frac{Y_{s}}{Y_{p}}\right)\right]/\left[1-\left(\frac{{\bar{Y}}_{s}}{{\bar{Y}}_{p}}\right)\right]$ (Fischer and Maurer, 1978).
(3) Mean Productivity

$(\text{MP})=\frac{Y_{p}+Y_{s}}{2}$ (Rosielle and Hamblin, 1981).
(4) Yield Stability index

$(\text{YSI})=Y_{s}/Y_{p}$ (Bouslama and Schapaugh, 1984).
(5) Drought resistance index

$(\text{DI})=\left[Y_{s}\text{x}\left(\frac{Y_{s}}{Y_{p}}\right)\right]/{\bar{Y}}_{s}$ (Farshadfar et al., 2012).

$Y_{p}$ and $Y_{s}$ were the grain yields of a genotype under control and drought conditions, respectively.

${\bar{Y}}_{p}$ and $\bar{Y_{s}}$ were the mean grain yields of all genotypes under non-stress and stress conditions, respectively.

### 2.11 qRT-PCR analysis

Two durum wheat genotypes (D117 and AG3) and two soft wheat genotypes (Utique and BT) were selected for this analysis. Dehulled seeds of wheat genotypes were surface sterilized as described by Bianco et al. (2021). Seeds were then washed several times with sterilized distilled water, positioned onto the surface of 0.8% water-agar plates and incubated at 21°C in the dark for germination. After 5 days, germinated seeds were transferred into plastic pots units (7 cm in length and 10 cm in diameter) containing sand (1.0 mm granule size) and perlite (3-4 mm granule size) soil in 3:1 ratio. Each planting unit was kept in the growth chamber under long daylight (16 h), 19–23°C temperature and 75% relative humidity and watered daily. Once a week a nitrogen-free nutrient medium (Bianco et al., 2021) was added to the plants. The 10-day-old plants were subjected to drought stress during 5 days by suspending water supply. After drought stress, leaves of both controls and treated plants were collected and used for the isolation of total RNA. Total RNA was extracted from 100 mg of frozen leaf tissues with Trizol Reagent (Sigma). Briefly, samples with Trizol were incubated for 10 min at 65°C before mixing with 0.2 mL chloroform (per mL Trizol). Samples were centrifuged at 12,000 x g for 15 min at 4°C, the upper aqueous phase was collected, and the RNA was precipitated with isopropanol. After the extraction was completed, RNA was resuspended in 20 μl RNase-free distilled H_2_O (Thermo Fisher Scientific). Residual DNA present in the RNA preparations was removed by using the TURBO DNA-free kit (Invitrogen™), according to the manufacturer’s instructions. RNA integrity was analyzed by native agarose gel electrophoresis, while the concentration and purity of the RNA samples were assessed using a NanoDrop spectrophotometer (Thermo Scientific). Total RNA (2 µg) was reverse transcribed using the Superscript^®^ III reverse transcription kit (Invitrogen™, Ca, USA). qRT-PCR with SYBR Green detection was performed. Relative mRNA expression levels were calculated according to the 2^–ΔΔ^Ct method (Livak and Schmittgen, 2001) using tubulin as endogenous control gene for the purpose of data normalization. Specific primers for the genes *PIP2:1* and *AAO* were as follows: *PIP2:1*, 5’-GGCCGGACTGAAGTGTAGAT-3’ and 5’-ACAGGACAAAGGTGTGGGAT-3’ (Yan et al., 2016); *AAO*, 5’-TTGGCGTTGTGATTGCTG-3’ and 5’-GCTCAAGGTTCTCGGTGCT-3’ (Ma et al., 2016). All reactions were assayed by using the Agilent AriaMX Real-Time system (Agilent Technologies), under the following cycling conditions: 95°C for 20 s, and 40 cycles at 95°C for 3 s and 60°C for 30 s. The results were analyzed using the Aria-MX software (version 1.6) and the relative fold change of gene expression was determined in drought stressed plants compared with the control ones. qRT-PCR data are the mean ± SD of three biological replicates and three technical replicates.

### 2.12 Statistical analysis

IBM Statistics SPSS 25 and OriginPro 2023 (Origin Lab) software were used for the statistical analysis. Data sets were presented as the means ± standard deviation (S.D.) of three replications for parameters derived from pressure-volume curves and five independent biological replicates for all the other traits. Two-way analyses of variance (ANOVA) were performed for all data, and the differences between the means were compared using Tukey’s multiple range test and indicated by different lowercase letters (p ≤ 0.05) (**Table S1**). The degree of correlation among the studied parameters was estimated using Pearson’s coefficient analyses. Principal component analysis (PCA) was employed to classify the stress tolerance indices into major components and assign rankings to the genotypes to obtain a more precise evaluation of the drought tolerance levels of wheat genotypes.

## 3 Results

Preliminary screening focused on the measurement of dry matter (DM) and grain yield (GY) of 28 Tunisian wheat genotypes grown under control and drought stress conditions revealed that the tested varieties showed very contrasting phenotypes (**Table 1**). Starting from these data we selected a total of 6 genotypes (4 durum wheat and 2 soft wheat) for further analyzes of drought stress resistance.

**Table 1:** Dry matter and grain yield of different wheat genotypes grown under control and drought stress conditions.

Variety	Dry Matter (g plant^-1^)	Drought (D)	RTC^*^	Grain Yield (g plant^-1^)	Drought (D)	RTC^*^
	Control (C)			Control (C)		
D117	1.22 ± 0.16 abc	0.61 ± 0.15 abcd	0.5	0.72 ± 0.05 abcdef	0.58 ± 0.02 ab	0.19
Agini	0.66 ± 0.05 bc	0.6 ± 0.07 abcd	0.1	0.77 ± 0.01 abcdef	0.15 ± 0.02 abcde	0.81
Biskri	1.73 ± 0.17 a	0.88 ± 0.07 abcd	0.49	0.67 ± 0.1 abcdef	0.04 ± 0.04 de	0.95
Inrat69	1.2 ± 0.13 abc	0.72 ± 0.13 abcd	0.4	0.8 ± 0.09 abcdef	0.04 ± 0.04 de	0.95
D-56-16-A Tunis ariana	1.15 ± 0.41 abc	0.8 ± 0.09 abcd	0.3	0.46 ± 0.03 cdef	0.24 ± 0.02 abcde	0.49
Maghrebi72	0.63 ± 0.1 c	0.29 ± 0.02 d	0.54	0.7 ± 0.1 abcdef	0.22 ± 0.02 abcde	0.69
BT	1.45 ± 0.32 abc	1.02 ± 0.14 abc	0.3	0.78 ± 0.07 abcdef	0.1 ± 0.02 cde	0.87
Syndiouk	1.52 ± 0.1 ab	1.2 ± 0.24 a	0.21	0.31 ± 0.02 def	0.38 ± 0.02 abcde	-0.25
Tunisian durum1	1.06 ± 0.08 abc	0.64 ± 0.07 abcd	0.39	0.97 ± 0.12 abc	0.24 ± 0.06 abcde	0.75
BD 1407-B	1.33 ± 0.23 abc	0.94 ± 0.14 abc	0.29	0.23 ± 0.05 f	0.17 ± 0.01 abcde	0.27
Amel72	0.97 ± 0.13 abc	0.48 ± 0.11 bcd	0.51	0.88 ± 0.07 abcd	0.55 ± 0.08 abc	0.38
Derbessi2	1.27 ± 0.3 abc	0.82 ± 0.02 abcd	0.35	0.35 ± 0.11 def	0.17 ± 0.03 abcde	0.52
Tunisian durum7	1.25 ± 0.05 abc	1.08 ± 0.01 ab	0.14	1.1 ± 0.06 ab	0.84 ± 0.1 a	0.23
Badri	1.1 ± 0.03 abc	0.79 ± 0.07 abcd	0.28	0.6 ± 0.22 bcdef	0.28 ± 0.15 abcde	0.53
D 58-25-A	1.15 ± 0.04 abc	0.53 ± 0.02 bcd	0.54	0.45 ± 0.01 cdef	0.05 ± 0.05 de	0.9
Mekki13	1.39 ± 0.02 abc	1.05 ± 0.15 ab	0.24	0.55 ± 0.03 bcdef	0.03 ± 0.03 de	0.94
Carthage74	0.96 ± 0.1 abc	0.54 ± 0.1 bcd	0.44	0.89 ± 0.1 abcd	0.54 ± 0.04 abc	0.39
Dougga74	1.12 ± 0.12 abc	0.67 ± 0.05 abcd	0.4	0.88 ± 0.16 abcd	0.32 ± 0.03 abcde	0.63
Florence-Aurore	1 ± 0.11 abc	0.79 ± 0.14 abcd	0.21	0.87 ± 0.14 abcd	0.43 ± 0.05 abcde	0.51
Ariana66	1.13 ± 0.28 abc	0.73 ± 0.07 abcd	0.35	0.56 ± 0.21 bcdef	0.15 ± 0.02 abcde	0.74
Utique	0.91 ± 0.03 abc	0.73 ± 0.16 abcd	0.2	1.2 ± 0.05 a	0.56 ± 0.25 ab	0.53
Zanzibar	1.52 ± 0.16 ab	0.98 ± 0.02 abc	0.35	0.69 ± 0.07 abcdef	0.13 ± 0.02 bcde	0.82
Mahmoudi AG3	0.98 ± 0.08 abc	0.79 ± 0.1 abcd	0.2	0.28 ± 0.03 ef	0.02 ± 0.02 e	0.94
Bidi490	1.25 ± 0.17 abc	0.86 ± 0.18 abcd	0.31	0.76 ± 0.15 abcdef	0.14 ± 0.02 abcde	0.81
Karim	0.77 ± 0.02 bc	0.44 ± 0.06 cd	0.43	0.62 ± 0.2 abcdef	0.59 ± 0.14 ab	0.05
Khiar	0.63 ± 0.01 c	0.55 0.15 bcd	0.14	0.83 ± 0.06 abcde	0.48 0.2 abcd	0.42
Maali	0.81 bc	0.48 ± 0.06 bcd	0.41	0.79 0.08 abcdef	0.36 0.08 abcde	0.55
Om rabii	0.75 bc	0.52 ± 0.06 bcd	0.3	0.75 0.04 abcdef	0.46 ± 0.08	0.39

In a column, the same letter(s) indicate non-significant differences, whereas distinct letters indicate significant differences (p< 0.05).

^*^Relative Trait Changes (RTC) were calculated as (Control-Drought)/Control.

### 3.1 Climatic parameters

**Figure 2:**
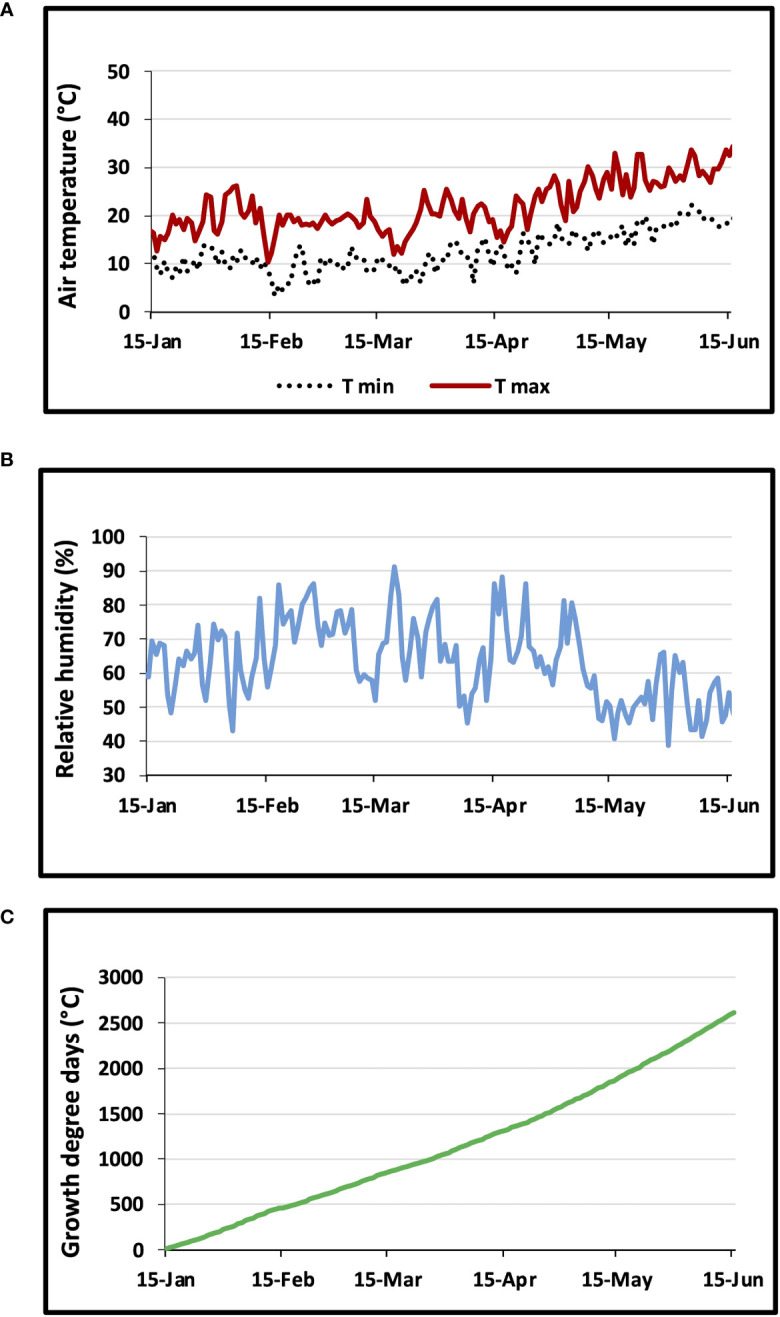
Changes in **(A)** daily maximum and minimum air temperature, **(B)** air relative humidity, and **(C)** growth degree days GDD.

**Figure 2** shows the daily variations in air temperature (**Figure 2A**), relative humidity (**Figure 2B**), and growth degree days (**Figure 2C**). From January to June, the monthly air temperature averages were 13, 14, 14, 17, 21 and 27°C, respectively. The average relative humidity values were about 62, 69, 70, 65, 57 and 53% for the same months. Throughout the wheat growing season, the GDD values reached 2615°C.

### 3.2 Water relation parameters

Water relations were determined after 3 weeks of drought treatment. Significant differences (p< 0.05) were observed for all measured parameters (**Table 2**). The analysis of P-V curves indicated that Ψπ^100^ and Ψπ^0^ decreased significantly in the genotypes D117, Syndiouk, Td7 and Utique in response to drought stress. The decrease in Ψπ^100^ and Ψπ^0^ was the highest in D117 and Syndiouk and intermediate in Td7 and Utique. However, both AG3 and BT didn’t show significant differences for these traits. Significant change in RWC_0_ was detected only for the genotype D117. After drought stress, the AWC increased in the genotypes D117, Syndiouk and Td7, at the rate of 18, 35 and 12%, respectively, as compared to the controls. In contrast, AG3 and BT showed a significant reduction in AWC by 21 and 14%, respectively, while no significant difference was observed with Utique genotype.

**Table 2:** Water relation parameters deriving from pressure-volume curves analysis in control and stressed wheat genotypes.

Genotype	Treatment	Ψπ^100^ (MPa)	ΔΨπ^100^ (OA)	Ψπ^0^ (MPa)	ΔΨπ^0^	RWC_0_(%)	AWC (%)	εmax (MPa)
**D117**	Control	-1.21 ± 0.08a		-1.81 ± 0.11ab		76.33 ± 0.94a	33.67 ± 1.53d	0.26 ± 0.02d
	Stressed	-1.67 ± 0.13b	0.46	-2.44 ± 0.15cd	0.63	80.67 ± 0.47a	39.67 ± 2.53bc	0.39 ± 0.03bc
**Syndiouk**	Control	-1.19 ± 0.10a		-1.85 ± 0.07ab		76.67 ± 3.09a	35.67 ± 1.15cd	0.31 ± 0.02d
	Stressed	-1.59 ± 0.15b	0.40	-2.59 ± 0.16d	0.74	78.67 ± 3.30a	48.00 ± 2.00a	0.61 ± 0.02a
**Td7**	Control	-0.98 ± 0.13a		-1.61 ± 0.24a		76.67 ± 0.47a	39.33 ± 0.07bc	0.32 ± 0.01cd
	Stressed	-1.21 ± 0.07a	0.23	-2.04 ± 0.08abc	0.43	76.33 ± 3.86a	44.00 ± 2.00ab	0.46 ± 0.03b
**AG3**	Control	-1.56 ± 0.16b		-1.99 ± 0.11abc		80.00 ± 3.64a	25.00 ± 2.00e	0.10 ± 0.01e
	Stressed	-1.65 ± 0.23b	0.09	-2.16 ± 0.11bcd	0.17	77.67 ± 3.30a	19.67 ± 1.53f	0.13 ± 0.01e
**Utique**	Control	-1.33 ± 0.08ab		-1.90 ± 0.06ab		81.33 ± 0.94a	42.67 ± 0.57b	0.29 ± 0.03d
	Stressed	-1.53 ± 0.17b	0.20	-2.16 ± 0.15bcd	0.26	78.50 ± 5.31a	42.00 ± 2.00b	0.45 ± 0.02b
**BT**	Control	-1.36 ± 0.06ab		-1.95 ± 0.13ab		78.67 ± 0.87a	42.33 ± 1.53b	0.33 ± 0.02cd
	Stressed	-1.49 ± 0.22ab	0.13	-2.13 ± 0.08bcd	0.18	78.33 ± 4.64a	36.33 ± 2.08cd	0.32 ± 0.02cd
P-value								
Genotype (G)		*p*< 0.01		*p<* 0.05		*p* > 0.05	*p*< 0.01	*p<* 0.001
Treatment (T)		*p*< 0.001		*p*< 0.001		*p* > 0.05	*p*< 0.001	*p*< 0.001
G * T		*p* > 0.05		*p*< 0.05		*p* > 0.05	*p*< 0.001	*p*< 0.001

Ψπ^100^, osmotic potential at full turgor; Ψπ^0^, osmotic potential at turgor loss point; RWC_0_, relative water content at turgor loss point; AWC, apoplasmic water content; εmax, bulk modulus of elasticity. Each value represents mean ± S.D (n = 3). Different letters within the same column indicates significant differences between treatments (P ≤ 0.05) according to Tukey’s test.

After exposure to drought stress, all genotypes, except BT, showed a significant increase (from 0.04 to 0.3 MPa) in Ɛmax, indicating a reduction in the elasticity of the cell wall. The BT genotype maintained similar values of Ɛmax under both control and drought stress conditions.

### 3.3 Organic solutes and ion accumulation

An increase in organic solutes (proline and soluble sugars), as well as inorganic ions (K^+^ and Ca^2+^), were observed in drought-stressed plants as compared to control ones (**Table 3**). All genotypes, except BT, displayed a significant increase in soluble carbohydrate levels. The accumulation of soluble sugars was particularly high in Td7 (76%) and D117 (73%), moderate in Utique (38%) and Syndiouk (33%). They were relatively low in AG3 (12%) and BT (3%), as compared to the controls.

**Table 3:** Solute concentrations (total soluble sugars, proline, K^+^, Na^+^ and Ca^2+^) in leaves of control and stressed wheat genotypes based on symplastic water content at full turgor.

Genotype	Treatment	Solute concentration (µmol g^-1^ symplastic water)					
		Total soluble sugars	Proline	K^+^	Na^+^	Ca^2+^	Total inorganic ions
**D117**	Control	345.9 ± 13.9ef	2.6 ± 0.4f	12.7 ± 0.1cd	18.1 ± 1.3a	7.5 ± 0.3bc	38.3
	Stressed	598.2 ± 45.6a	3.5 ± 0.2ef	15.3 ± 1.1bc	20.6 ± 1.8a	8.4 ± 0.8ab	44.4
**Syndiouk**	Control	343.4 ± 9.5f	2.9 ± 0.3ef	15.5 ± 1.5bc	18.2 ± 1.5a	8.3 ± 0.6bc	41.9
	Stressed	472.4 ± 21.0bc	7.6 ± 0.9a	21.2 ± 0.9a	24.6 ± 2.2a	10.1 ± 1.0a	55.9
**Td7**	Control	229.6 ± 14.4g	6.3 ± 0.2abc	18.2 ± 0.6ab	19.1 ± 2.4a	7.6 ± 0.5bc	44.9
	Stressed	405.2 ± 30.1de	7.0 ± 0.8ab	20.0 ± 1.3a	22.0 ± 2.6a	7.1 ± 0.2bc	49.0
**AG3**	Control	272.7 ± 17.3g	4.7 ± 0.5cde	10.9 ± 0.7d	16.7 ± 1.4a	4.6 ± 0.5c	32.2
	Stressed	306.5 ± 13.5ef	4.1 ± 0.3def	12.4 ± 1.0cd	17.0 ± 0.9a	4.6 ± 0.3c	28.7
**Utique**	Control	375.8 ± 14.3def	5.6 ± 0.4bcd	18.9 ± 1.6ab	22.0 ± 1.7a	7.7 ± 1.1bc	48.6
	Stressed	498.2 ± 34.9b	6.9 ± 0.4ab	16.7 ± 0.7bc	20.3 ± 1.5a	7.9 ± 0.8bc	44.8
**BT**	Control	445.9 ± 31.8cd	4.6 ± 0.5cde	17.3 ± 1.2ab	18.9 ± 0.8a	6.5 ± 0.6bc	42.7
	Stressed	460.8 ± 29.5c	7.0 ± 0.8ab	14.2 ± 0.8bc	19.2 ± 1.4a	7.0 ± 0.8bc	40.4
P-value							
Genotype (G)		*p*< 0.001	*p*< 0.001	*p*< 0.001	*p* > 0.05	*p*< 0.001	
Treatment (T)		*p*< 0.001	*p*< 0.001	*p* > 0.05	*p* > 0.05	*p* > 0.05	
G * T		*p*< 0.001	*p*< 0.001	*p*< 0.05	*p* > 0.05	*p* > 0.05	

Each value represents mean ± S.D (n = 5). Different letters within the same column indicates significant differences between treatments (P ≤ 0.05) according to Tukey’s test.

After drought stress, a significant increase in leaf proline content was observed for the genotypes Syndiouk (up to 162%) and BT (up to 52%). These findings suggest that the enhanced accumulation of proline in wheat plants is not specifically linked to a particular tolerance strategy.

Drought stress led to a significant accumulation of K^+^ and Ca^2+^ only in the leaves of Syndiouk (**Table 3**). Furthermore, no significant differences were observed for the sodium (Na^+^) content in the leaves of stressed and control plants.

### 3.4 Contribution of solutes to osmotic adjustment

Leaf total soluble sugars played the most prominent role in osmotic potential variations at full turgor (Ψπ^100^). On the contrary, proline did not contribute significantly to the osmotic adjustment of wheat plants subjected to water stress. The greatest impact of inorganic ions on osmotic adjustment was observed for the genotype Syndiouk. Our data indicates that, under drought stress, the osmotic adjustment mechanism in wheat leaves was mainly attributed to the accumulation of carbohydrates (**Table 4**).

**Table 4:** Contribution of solutes to osmotic adjustment in stressed wheat leaves.

Genotype	Contribution to osmotic adjustment (%)		
	Total soluble sugars	Proline	Total inorganic ions
**D117**	137.1	0.5	3.3
**Syndiouk**	80.6	2.9	8.7
**Td7**	190.8	0.7	4.4
**AG3**	93.7	-1.6	-9.7
**Utique**	153.0	1.5	-4.7
**BT**	67.1	0.6	-4.5

### 3.5 Gas exchange and water use efficiency

**Figure 3:**
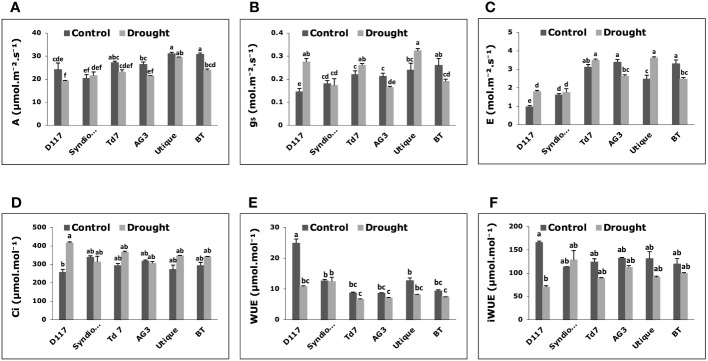
Variations of gas exchange parameters in wheat genotypes grown under control and drought stress conditions. **(A)** Net photosynthesis (A); **(B)** stomatal conductance (gs); **(C)** transpiration rate (E); **(D)** intercellular CO_2_ concentration (Ci); **(E)** water use efficiency (WUE); **(F)** intrinsic water use efficiency (iWUE). Data are the means ± S.D (n = 5). Means with different letters are significantly different at 5% level of confidence (P ≤ 0.05) according to Tukey’s test.

After drought stress, significant changes in gas exchange parameters were observed for all selected wheat genotypes except for Syndiouk (**Figure 3**). The net photosynthesis rate (A) did not show a significant difference in Syndiouk, Td7, and Utique as compared to the controls, while it was significantly reduced for genotypes D117, AG3, and BT (**Figure 3A**). Under stress conditions, stomatal conductance (**Figure 3B**) and transpiration rate (**Figure 3C**) were higher in D117 and Utique, unchanged in Syndiouk, and significantly decreased in AG3 and BT plants.

Moreover, an elevated level of intercellular CO_2_ (Ci) was observed for the genotype D117 after the drought treatment (**Figure 3D**). WUE and iWUE are important parameters indicating how efficiently plants use water. All genotypes showed no variation in WUE and iWUE values as compared to the controls (**Figures 3E, F**), except for D117, which exhibited a significant decrease in both parameters under drought stress conditions.

### 3.6 Leaf area, stomatal density and total chlorophyll content

**Figure 4:**
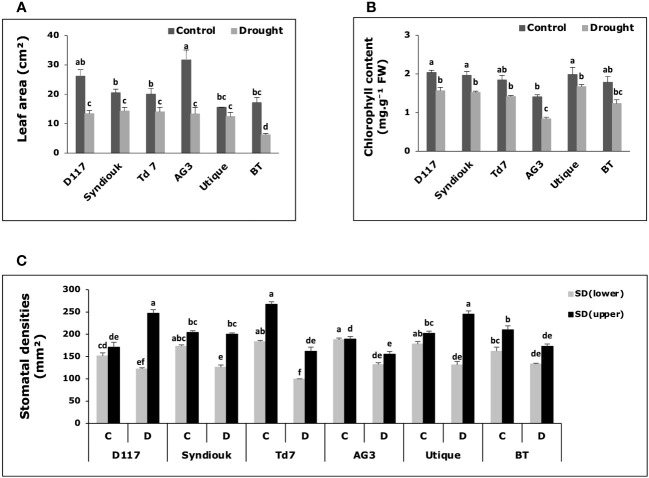
Variations of physiological parameters in wheat genotypes grown under control (C) and drought stress conditions (D). **(A)** Leaf area; **(B)** total chlorophyll content; **(C)** stomatal density on lower leaf surface (SD_lower_) and upper leaf surface (SD_upper_). Data are the means ± S.D (n = 5). Means with different letters are significantly different at 5% level of confidence (P ≤ 0.05 according to Tukey’s test).

Drought stress led to a decrease in plant leaf area (**Figure 4A**), total chlorophyll content (**Figure 4B**) and stomatal density on the lower surface (SDlower) for all genotypes (**Figure 4C**). These reductions were particularly pronounced for AG3 and BT, with a decrease in leaf area of about 60%. Chlorophyl content decreased in AG3 and BT by approximately 41% and 31%, respectively. On the contrary, a significant increase in stomatal density on the upper leaf surface (SDupper) was observed for the genotypes D117 and Utique after drought stress (**Figure 4C**).

### 3.7 Lipid peroxidation and membrane permeability

**Figure 5:**
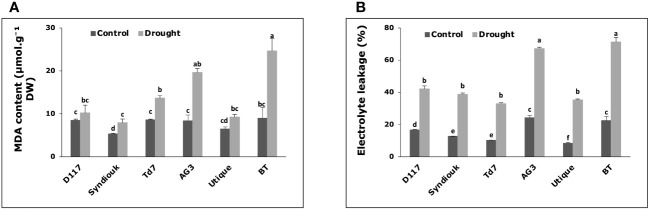
Variations of biochemical parameters in wheat genotypes grown under control and drought conditions. **(A)** malondialdehyde (MDA) content; **(B)** electrolyte leakage. Data are the means ± S.D (n = 5). Means with different letters are significantly different at 5% level of confidence (P ≤ 0.05 according to Tukey’s test).

Enhanced levels of MDA (malondialdehyde) were observed in all plants subjected to drought stress, except for D117 and Utique, for which no significant differences were measured between contrl and stressed plants (**Figure 5A**). BT and AG3 stressed plants exhibited the highest MDA levels (2.3-fold and 2.7-fold increase, respectively) as compared to the control plants. Simultaneously, drought stress led to a significant increase in the leakage rate of cellular electrolytes in the leaves of all studied genotypes (**Figure 5B**). The greatest effect was observed for AG3 (2.8-fold increase) and BT (3.2-fold increase) stressed plants as compared to the control ones.

### 3.8 Yield components and drought tolerance indices

**Figure 6:**
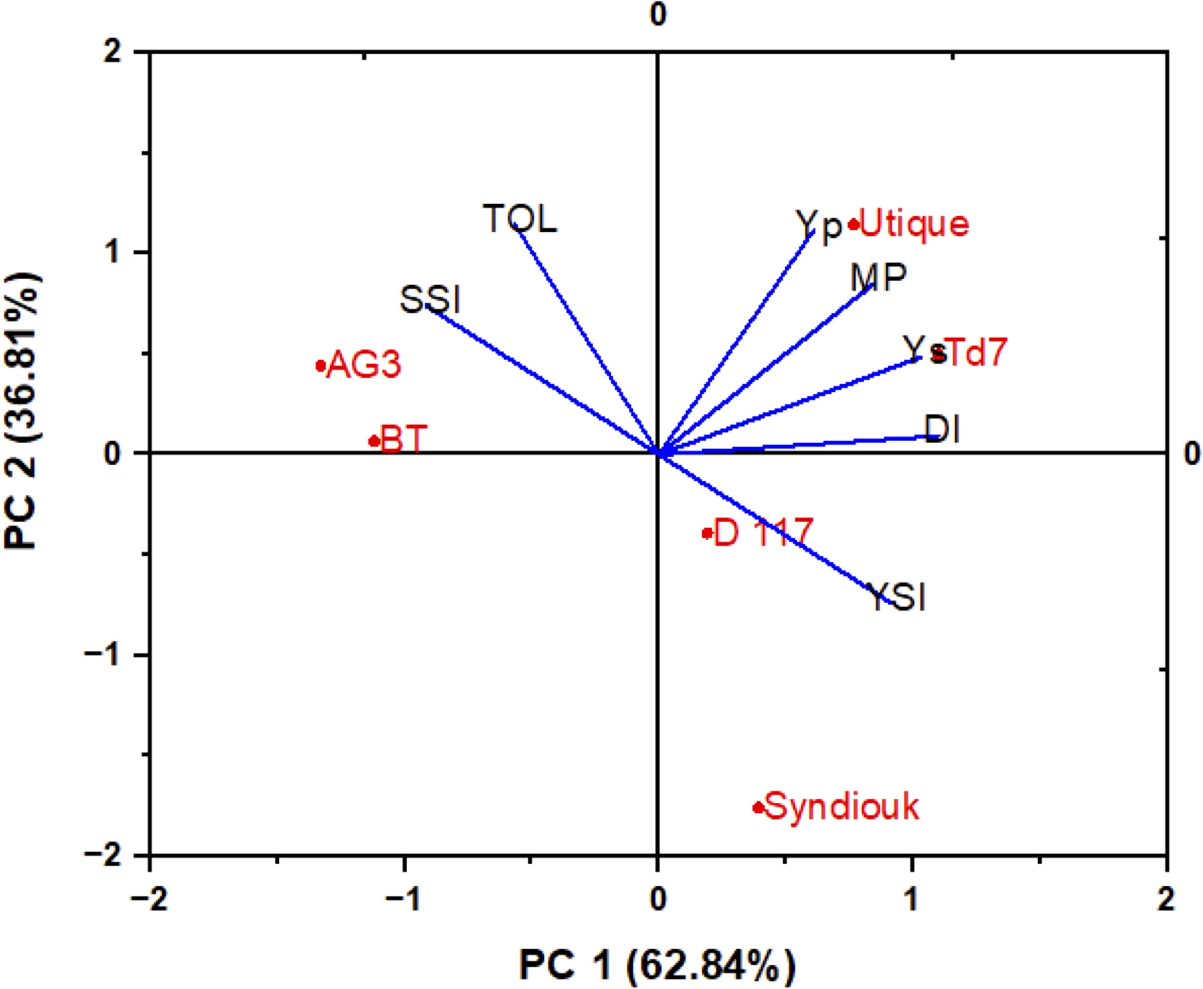
Biplot of principle components analysis of six wheat genotypes based on drought tolerance indices.

Drought stress led to a significant reduction of all yield parameters (**Table 5**). Almost all tested genotypes showed a reduction in biological yield per plant (BY). D117 was the least affected genotype, with only 11% reduction, while AG3 and BT were the most severely impacted, with 50% reductions. The greatest decrease in grain yield (GY) was observed for AG3 (69%) and BT (61%) as compared to the control plants. However, Syndiouk and Utique did not exhibit a significant effect on GY. In addition, the number of seeds per plant was reduced in AG3 and BT up to 58% and 40%, respectively. The weight of 1000 seeds was also reduced by about 35% for both genotypes. Overall, Td7 and Utique showed the highest yield attributes under drought stress conditions. A biplot of principle components analysis (PCA) was developed from the first two principal components (PC1 and PC2) to classify the six varieties according to the drought tolerance indices (**Figure 6**). PC1 accounted for the largest variance, explaining approximately 62.84% of the total variation, while PC2 accounted for 36.81% of the total variation. Together, PCA1 and PCA2 represented 99% of the total variation. The genotypes were classified into three groups (A, B and C); The group A comprised the most productive genotypes in both stress and non-stress conditions (Utique and TD7). These varieties exhibited positive values for both PC1 and PC2, indicating their proximity to traits such as MP (mean productivity), Yp (yield potential), and Ys (yield stability). The group B represented the most drought-tolerant varieties (D117 and Syndiouk) displaying consistent performance. These genotypes had negative values for PC1 and positive values for PC2, indicating their proximity to indices such as DI (drought intensity) and YSI (yield stability index). The group C included the most drought-sensitive genotypes (AG3 and BT), which exhibited positive values for both PC1 and PC2. These genotypes were closer to indices such as TOL (tolerance) and SSI (susceptibility) in the biplot.

**Table 5:** Biological yield, grain yield, number of seeds per plant and weight of 1000 seeds in six wheat genotypes under control and drought stress conditions.

Genotype	Treatment	Biological yield (g/plant)	Grain yield (g/plant)	Number of seeds/plant	Weight of 1000 seeds (g)
**D117**	Control	6.42 ± 0.54cdef	2.83 ± 0.16cd	84.8 ± 2.23cd	33.42 ± 2.23a
	Stressed	5.70 ± 0.41f	2.03 ± 0.19cde	90.0 ± 19.9cd	23.50 ± 4.30bc
**Syndiouk**	Control	9.64 ± 0.71a	1.71 ± 0.44de	138.2 ± 20.75ab	12.14 ± 1.61d
	Stressed	8.41 ± 0.38ab	1.65 ± 0.35de	119.4 ± 17.53abcd	13.76 ± 1.64d
**Td7**	Control	7.60 ± 0.84bcde	4.16 ± 0.63ab	127.2 ± 27.95abc	33.27 ± 3.43a
	Stressed	6.15 ± 0.09def	3.19 ± 0.28bc	96.4 ± 4.45bcd	33.22 ± 3.75a
**AG3**	Control	8.02 ± 0.99abc	2.67 ± 0.19cd	90.4 ± 8.55cd	29.72 ± 2.91ab
	Stressed	3.96 ± 0.48g	0.82 ± 0.07e	37.6 ± 22.50e	19.29 ± 5.74cd
**Utique**	Control	7.90 ± 1.11bc	4.63 ± 1.04a	140.0 ± 15.96a	32.79 ± 4.98a
	Stressed	5.99 ± 0.68ef	3.05 ± 0.65bc	125.6 ± 6.08abc	24.45 ± 5.65abc
**BT**	Control	7.76 ± 0.96bcd	2.44 ± 0.11cd	134.4 ± 26.31ab	18.81 ± 3.18cd
	Stressed	3.96 ± 0.51g	0.95 ± 0.20e	80.0 ± 16.49de	12.01 ± 0.90d
P-value					
Genotype		*p*< 0.001	*p*< 0.001	*p*< 0.001	*p*< 0.001
Treatment		*p*< 0.001	*p*< 0.001	*p*< 0.001	*p*< 0.001
G*T		*p*< 0.001	*p*< 0.05	*p*< 0.05	*p*< 0.01

Each value represents mean ± S.D (n = 5). Different letters within the same column indicates significant differences between treatments (P ≤ 0.05) according to Tukey’s test.

### 3.9 Correlations between the studied parameters

**Figure 7:**
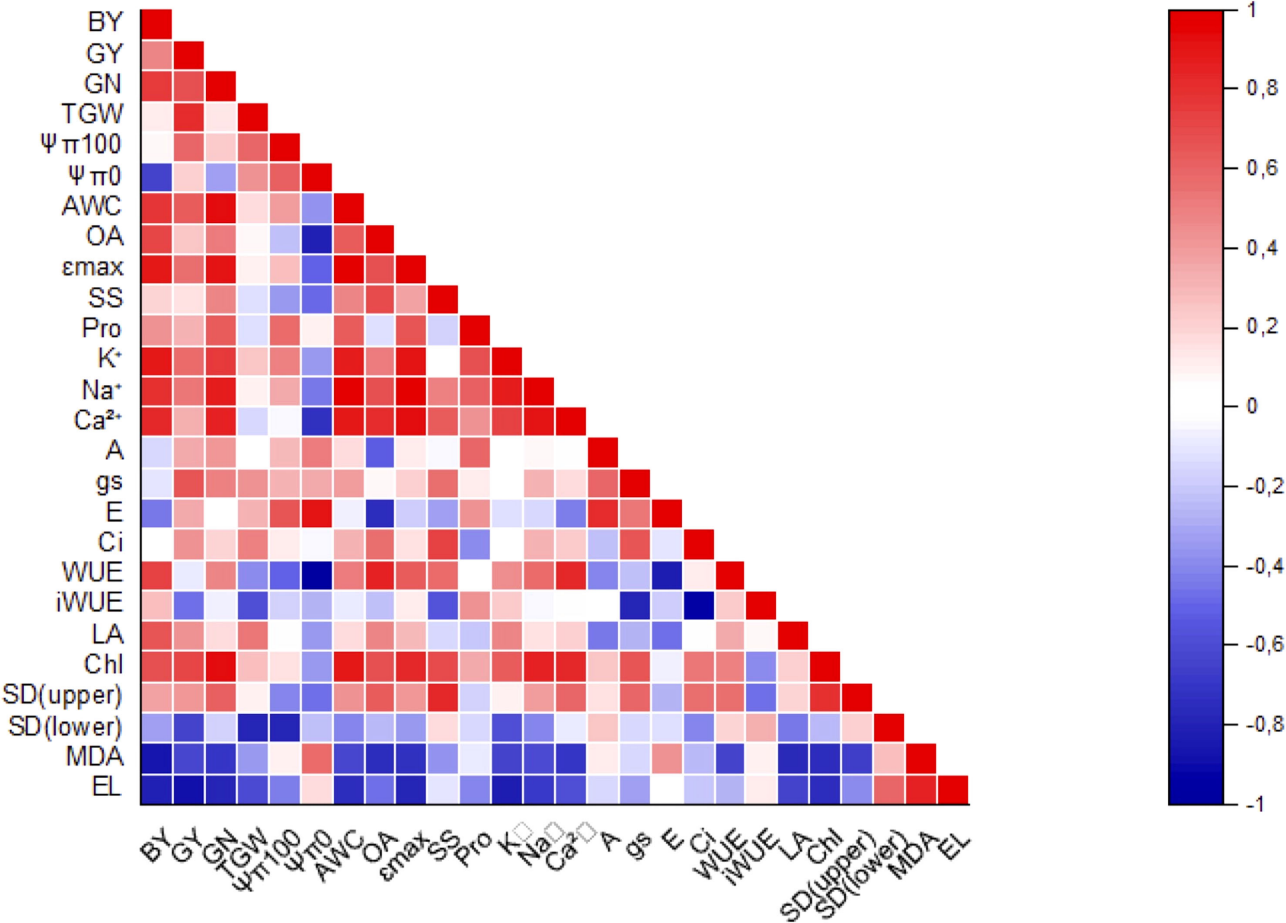
Correlation plot of measured parameters. Correlations were scaled from 1.0 to -1.0. The blue and red colors indicated positive and negative correlations, respectively.

Significant positive correlations were observed between leaf inorganic ions, total chlorophyll content, yield components (BY, GN) and water relation parameters (AWC, Ɛmax) (**Figure 7**). AWC and Na+ were highly correlated (p ≤ 0.001). On the contrary, significant negative correlations were detected between electrolyte leakeage (EL) and GY, EL and K+, MDA and BY, and iWUE and Ci. WUE and Ψπ0 were highly correlated (p ≤ 0.001).

### 3.10 Transcriptional analysis of drought-responsive genes

**Figure 8:**
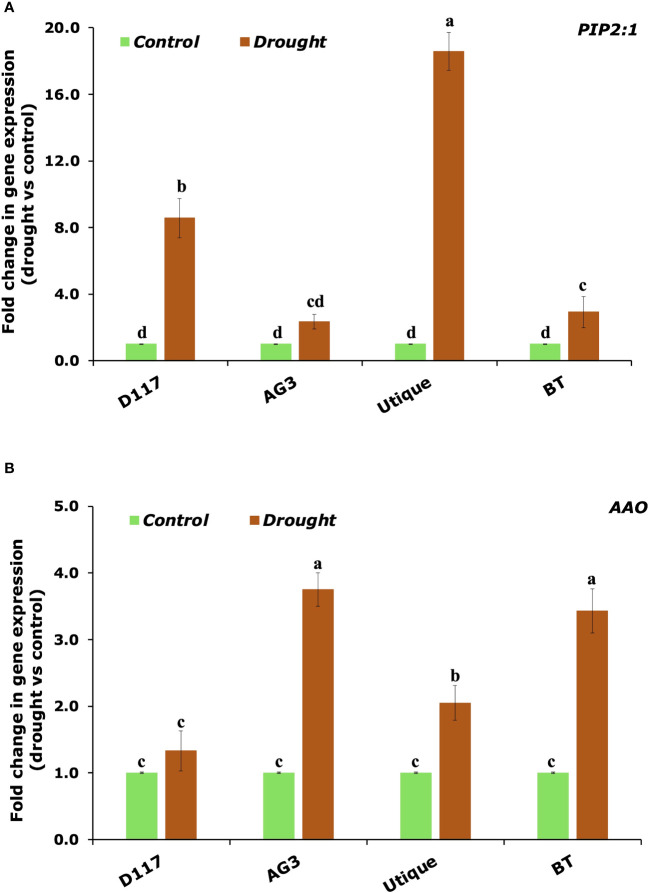
qPCR analysis of the genes involved in plant response to drought stress. Quantitative measurement of the PIP2:1 **(A)** and AAO **(B)** transcript levels in durum (D117 and AG3) and soft (Utique and BT) wheat plants subject to drought stress. Values are the means ± SD of three different biological replicates and three technical replicates. Means with different letters are significantly different at 5% level of confidence (P ≤ 0.05 according to Tukey’s test).

qRT-PCR analysis was performed to evaluate the effect of drought stress on the expression levels of selected genes involved in plant water uptake and in the biosynthesis of the main drought stress messenger, ABA. For this analysis, the four genotypes (two for durum wheat and two for soft wheat) showing the most contrasting responses to drought stress were selected. Data reported in **Figure 8** show that the drought stress induced the expression of the aquaporin PIP2:1 in all the selected genotypes. However, the triggering effect was stronger for the genotypes D117 and Utique. An opposite result was observed for the expression of the AAO gene, which catalyzes the final step of ABA biosynthesis: this gene was significantly induced in the genotypes AG3 and BT, while it was only slightly induced in the genotypes D117 and Utique.

## 4 Discussion

Drought stress poses a serious threat to crop productivity. Reduced soil water availability strongly affects several aspects of plant physiology leading plants to adjust their water balance. Water related parameters are among the first physiological traits that are affected by osmotic stress during the vegetative phase in Damask rose (Al-Yasi et al., 2020) and Spartina alterniflora (Hessini et al., 2009). Our findings indicated that the selected wheat genotypes responded to drought stress by activating an adaptive mechanism that involved the maintenance of cell turgor through osmotic adjustment (OA) and cellular elasticity (Ɛmax) (**Table 2**). At the end of the drought treatment, stressed plants of the tolerant genotypes D117 and Syndiouk exhibited a significant higher apoplasmic water content (AWC) compared to control plants. This adaptation could contribute to the accumulation of solutes and thereby could help maintaining turgor in stressed leaves (Hessini et al., 2008). Accordingly, D117 and Syndiouk displayed a decrease in leaf osmotic potential at full turgor (Ψπ^100^) and at turgor loss point (Ψπ^0^), while leaf relative water content (RWC_0_) did not change significantly.

Based on the findings of Martínez et al. (2007), we suggest that the increase in apoplastic water reserves and the decrease in Ψπ were sufficient to prevent significant water loss. In addition to the decrease of the Ψπ, drought stress induced a reduction in cell walls elasticity. Both increase and decrease in the bulk modulus of elasticity (Ɛmax) have been described as adaptive responses to drought stress (Leuschner et al., 2019). The increase in Ɛmax results in stiffer cell walls (Juenger and Verslues, 2023). In almost all genotypes (**Table 2**), higher Ɛmax facilitated water extraction creating a greater gradient in water potential from the soil to the leaves (Hessini et al., 2008). This gradient promoted a more efficient water uptake during period of high transpirational demand (**Figure 2**). Our findings agree with previous study on plants subjected to drought stress (Patakas et al., 2002; Martínez et al., 2007; Hessini et al., 2008). Syndiouk, D117, Td7, and Utique genotypes exhibited a significant increase in Ɛmax, with the highest increase observed for Syndiouk. On the other hand, AG3 and BT did not show statistically significant changes in cell wall extensibility. These results indicate that the alterations in the mechanical properties of cell walls in AG3 and BT were mainly influenced by their distinct behavior, which differed significantly from the other genotypes. An increase in hemi-cellulose content of the cell membrane has been reported in wheat plants exposed to drought stress (Wakabayashi et al., 1997). In addition, genotypes exhibiting active osmotic adjustments and accumulating high levels of solutes after drought stress maintained cell integrity through inelastic cell walls (Patakas et al., 2002).

Our results showed that plants ability to adjust the cell osmolarity and membrane elasticity under drought stress was related to higher cell membrane stability. The significant increase in Ɛmax and the higher osmotic adjustment values recorded for the genotypes D117, Syndiouk, Td7, and Utique (**Table 2**) were associated with lower levels of malondialdehyde (MDA) (**Figure 5A**) and lower increase in electrolyte leakage (**Figure 5B**). The osmotic changes (ΔΨπ) observed for the selected genotypes after drought stress indicated an active accumulation of solutes in the leaves. Soluble sugars play a key role in osmotic adjustment (Abeed and Dawood, 2020), and drought stress alters carbon assimilation in response to a photosynthetic rate inhibition (Patakas et al., 2002). However, in our study, leaf carbohydrate levels did not decrease in stressed plants. On the contrary, we observed an accumulation of these organic solutes in drought-stressed wheat plant leaves. This accumulation resulted from the inhibition of assimilate production, not strong enough to compensate for its consumption (Quick et al., 1992). It has been shown that carbohydrate and/or sugars deriving from starch degradation accumulated in the leaves of stressed plants and could be transferred from mature to growing leaves, thus contributing to osmotic adjustment (Patakas et al., 2002).

Compared to soluble sugars, the contribution of proline to the osmotic adjustment was not significant (2.9%) (**Table 4**). In addition, there was no clear correlation between proline accumulation and specific drought response strategy, indicating that it cannot be considered as a reliable selection criterion for drought tolerance in the studied wheat genotypes. Sanchez et al. (2004) reported that, in response to drought stress, proline mainly accumulates in the symplast and therefore, in addition to its contribution to osmotic adjustment it may have a more complex role in conferring osmotic resistance. Furthermore, the accumulation of different ions during drought is of great interest. In our study, potassium (K^+^) and calcium (Ca^2+^) content significantly increased only in Syndiouk stressed plants (**Table 3**). These ions can be used as economic and efficient osmotic regulators to improve water status and increase drought resistance. Ca^2+^ ions stimulate photosynthesis, enhance nutrient uptake and control water use efficiency (Patakas et al., 2002; Cui et al., 2019; Huihui et al., 2021). Furthermore, calcium is generally believed to enhance cell wall rigidity and its protection against oxidative damage (Patakas et al., 2002). These effects could explain the significant increase in bulk modulus of elasticity (Ɛmax) (**Table 2**), the reduced levels of malondialdehyde (MDA) and the lower electrolyte leakage (EL) rates observed for Syndiouk under water stress (**Figure 5**).

Since the content of inorganic ions in the leaves of other wheat varieties did not increase under drought stress (**Table 3**), it is possible that other solutes, such as organic acids, glycine betaine and asparagine, could be involved in the osmotic adjustment of these two genotypes (Hessini et al., 2008). Our data showed that organic solutes, especially carbohydrates, played a significant role in osmotic adjustment of wheat genotypes (**Table 4**). These findings are consistent with the results of Bhutto et al. (2023) who reported that under drought stress, the increase in soluble carbohydrate content enabled wheat plants to lower their water potential and to protect cell membranes, soluble proteins and phospholipids.

In our study, the consistent accumulation of soluble sugars under water deficit conditions could be considered one of the key mechanisms adopted by wheat plants to tolerate drought stress. This strategy enhanced the capacity for osmotic adjustment, improved water preservation, regulated stomatal behavior, facilitated CO_2_ fixation, and stabilized macromolecules involved in photosynthetic efficiency and stress tolerance (Abeed and Dawood, 2020).

Regarding changes in water relations and osmolyte accumulation, our results indicate that the six selected wheat genotypes employed distinct strategies to cope with stress. The genotypes AG3 and BT, exhibited an isohydric response, as evidenced by their smaller differences in Ψπ^100^ and Ψπ^0^ between the control and stressed conditions compared to the other genotypes, and a decrease in transpiration rate (E), suggesting an effective stomatal control to maintain internal water balance (Onyemaobi et al., 2021).

Due to the crucial role of stomata in gas exchange, their closure during drought stress can have a negative impact on CO_2_ diffusion, and subsequently on photosynthetic rate (Pitaloka et al., 2022). Under drought stress, the repression of photosynthetic activity observed for the genotypes AG3 and BT was not only due to the reduced stomatal conductance but also to a more pronounced decrease in leaf area (**Figure 4A**) and chlorophyll content (**Figure 4B**). This decline was associated with a decrease in stomatal density on both the lower and upper leaf surfaces (**Figure 4C**), indicating that under such drought stress, photosynthesis was constrained by both stomatal and non-stomatal limitation in AG3 and BT genotypes (Flexas et al., 2006). The inhibition of chloroplast activity (Hasanuzzaman et al., 2023) led to an excessive amount of excitation energy in chloroplasts, which destroyed the equilibrium of electron transfer reactions, leading to the accumulation of highly reactive oxygen species (Liu et al., 2022) and the over-production of malondialdehyde (MDA). In absence of osmoprotectants, the high level of MDA content and the increase in electrolyte leakage observed for AG3 and BT stressed plants led to dysfunction of the cell membranes (Abeed and Dawood, 2020). In drought stress conditions, the preservation of water is the main strategy employed by the genotypes AG3 and BT to minimize water loss. However, this approach resulted in a premature shut down of physiological activity, which could explain the relatively high sensitivity of yield components to drought observed for these two genotypes as compared to other ones.

Anisohydric varieties adopt a different approach. They exhibit higher ΔΨπ^0^ values and accumulate osmotic compounds, which results in a greater adjustment of membrane elasticity and cell osmolarity. For the genotypes D117, Syndiouk, Td7 and Utique, a more pronounced decrease in Ψπ^0^ was observed. This characteristic enabled these plants to absorb water from drying soil even under limited water availability conditions (Leuschner et al., 2019). Consequently, in these genotypes cells could maintain turgor for a longer time before reaching the point of turgor loss (Hachani et al., 2022).

This finding was in line with the increase in cell membrane rigidity observed for these plants (**Table 2**). The thicker cell walls also play a compensatory role in counteracting the effect of reduced Ψπ (Patakas et al., 2002). The enhanced rigidity of leaf tissue and the ΔΨπ values measured in the selected genotypes allowed the maintenance of open stomata, thereby facilitating dynamic gas exchange. This phenomenon contributed to the preservation of stomatal conductance (gs) and photosynthetic assimilation (A), as illustrated in **Figure 3** (Hachani et al., 2022). Among the selected genotypes studied, Syndiouk and Utique exhibited the highest A rate maintenance under drought conditions, while D117 showed the highest gs. This behavior of anisohydric plants highlighted their acclimatization ability during periods of stress, as evidenced by the growth parameters measured (Gallé et al., 2013).

Stomatal functioning can be influenced by hormonal or morphological behavior resulting in higher gs values. To further analyse this aspect, qRT-PCR analysis was carried out to measure the expression levels of the gene AAO involved in the last step of ABA biosynthesis and the gene PIP2:1, encoding for a plasma membrane intrinsic protein (PIP), which is part of the aquaporin family. We showed that the genotypes exhibiting higher gs values (D117 and Utique) were those in which AAO gene for ABA biosynthesis was only slightly induced after drought stress, allowing stomata to remain open for a longer period. The lack of correlation between stomatal conductance (gs) and photosynthesis (A) observed for the genotype D117 could arise from the low induction of gene AAO involved in ABA biosynthesis, which allowed stomata to remain open for a longer period. However, even if an adequate supply of CO_2_ was ensured by the increase in stomatal conductance, the efficiency of photosynthesis could still be hampered by other limiting factors (such as slow activation of electron transport, key enzymes such as Rubisco in the Calvin-Benson, and the synthesis of sucrose) (Stitt and Schreiber, 1988; Carmo-Silva and Salvucci, 2013; Yamori, 2016; Kaiser et al., 2016). Consistent with our data, results reported in von Caemmerer et al. (2004) revealed that stomatal conductance was not strictly correlated with the photosynthetic capacity of guard cells or leaf mesophyll.

Moreover, drought stress triggered a strong up-regulation of the gene PIP2:1 in the same genotypes. This confirmed that the overexpression of PIP2:1 could enhance not only water movement across the cell membrane, but also gas exchange, as PIP2:1 is a CO_2_-permeable aquaporin (Wang et al., 2016). Our results are consistent with the findings of Sade et al. (2009), who reported that SlTIP2:2 overexpression limited the reduction in transpiration under drought stress, which ensured continuous CO_2_ uptake, promoting plant growth and yield production. Based on these findings, we suggest that: i) an elevated ABA content may have led to a transcriptional down-regulation of aquaporins, and ii) the variations in aquaporin transcript levels in response to drought could be linked to a divergent root hydraulic conductivity between isohydric and anisohydric wheat genotypes (Coupel-Ledru et al., 2017).

Furthermore, D117, Syndiouk, Td7, and Utique displayed elevated MDA contents in leaves (**Figure 5A**) and maintained stable yield levels following the stress period (**Table 5**). The accumulation of MDA under drought stress could indicate an adaptive mechanism. It has been reported that to maintain gs in the absence of ABA hormone repression, MDA might act as a signal for the expression of genes involved in the synthesis of enzymes and antioxidant molecules (Gallé et al., 2013; Onyemaobi et al., 2021). This suggestion is consistent with recent studies demonstrating that leaves are the primary site for ABA biosynthesis (Cardoso et al., 2020). Moreover, D117 and Utique were able to adjust their stomatal densities to optimize the functioning of the photosynthetic machinery. They reduced the stomatal density on the lower leaf surface (SDlower), thereby limiting transpiration, and compensated this reduction by increasing the number of stomata on the upper leaf surface (SDupper) (**Figure 4C**).

Our study showed that, among the yield components, Grain yield (GY) was the most adversely affected by the drought treatment (**Table 5**). The reduction was particularly pronounced in the genotypes AG3 and BT, which showed a decrease of 69% and 61%, respectively.

In contrast, Syndiouk was the least affected by water stress, as its GY, seed number, and the weight of 1000 seed were unchanged. On the other hand, the AG3 and BT genotypes produced significantly fewer and lighter seeds following drought treatment, as indicated by the decrease in 1000 seed weight, suggesting a decline in seed quality (**Table 5**).

The analysis of drought tolerance indices allows the classification of wheat varieties based on their productivity under normal and stressful conditions. The stress susceptibility index (SSI) and stress tolerance (TOL) are used to evaluate drought-tolerant genotypes able to achieve high yields under both normal and stress conditions (Mardeh et al., 2006). High values of these indices mean high susceptibility to drought stress (Fischer and Maurer, 1978; Rosielle and Hamblin, 1981). The genotypes AG3 and BT exhibited the highest TOL and SSI values. In addition, the high mean productivity (MP) values indicated a high productivity even in extreme environments. In the principal components analysis (**Figure 6**), the Utique and Td7 genotypes were close to Yp, Ys, and MP, demonstrating their high performance in terms of grain yield production under both control and drought conditions. The drought resistance index (DI) and yield stability index (YSI) are suitable parameters for selecting the most drought-tolerant genotypes (Khare et al., 2022). The D117 and Syndouk genotypes displayed the highest DI and YSI values, and the lowest TOL and SSI indices, suggesting a stable yield production due to their higher drought resistance. Based our results, the genotypes Syndiouk, D117, Td7 and Utique genotypes were classified as drought-tolerant, while AG3 and BT were categorized as sensitive.

## 5 Conclusion

The findings of the present study highlighted the relationship between drought tolerance and specific physio-molecular adaptations in the selected wheat genotypes. These adaptations included the adjustment of osmotic potential, the enhancement of cell wall rigidity and the maintenance of photosynthetic activity through the regulation of stomatal behavior. We suggest that measures such as ΔΨπ, Ɛmax and gs could be used as primary selection criteria for the robust screening of numerous genotypes in wheat breeding programs. Further exploration of the molecular mechanisms underlying the proposed differentiation between isohydric and anishydric genotypes showed that the overexpression of the PIP2:1 gene and the absence of induction of AAO gene expression under water-stressed conditions represent potential indicators for preliminary selection, even at a very early stage, of drought-tolerant genotypes.

Based on the overall responses of the six studied genotypes, Syndiouk, D117, Utique, and Td7 could be promising for future wheat breeding programs aimed at developing high-yielding and drought-tolerant varieties.

## Data availability statement

The original contributions presented in the study are included in the article/**Supplementary Material**. Further inquiries can be directed to the corresponding authors.

## Author contributions

AG, CB, HA, and FG designed the project and the strategy. HA, MA, contributed to plant sample collection and processing. AG, CB, FG, HA wrote and revised the manuscript. NR and MF-S were involved in the revision of the manuscript. NR and MF-S performed the molecular analysis. EB and RD helped with a critical discussion on the work. All authors contributed to the article and approved the submitted version
