# Supplementary material for: Drought responsiveness in six wheat genotypes: identification of stress resistance indicators
Source: Front Plant Sci. 2023 Sep 13;14:1232583. doi: 10.3389/fpls.2023.1232583 (PMC10534941; doi:10.3389/fpls.2023.1232583)
Supplement: Supplementary file 1 [file DataSheet_1.pdf]

Supplementary Material

Drought responsiveness in six wheat genotypes: identification of stress resistance indicators

Asma Guizani[1*], Hend Askri[2], Maria Laura Amenta[3], Roberto Defez[3], Elyes Babay[4,5], Carmen Bianco[3*], Nicoletta Rapana[6], Mariella M Finetti-Sialer[6], Fatma Gharbi[1]

[1]Laboratory of Mycology, Pathologies and Biomarkers LR16ES05, Faculty of Sciences of Tunis, 2092 Tunis, University of Tunis El Manar, Tunis, Tunisia;

[2]Laboratory of Valorization of Non-Conventional Water (LR16INRGREF02), National Institute of Rural Engineering, Water and Forestry, Carthage University, Tunis 1004, Tunisia;

[3]Institute of Biosciences and BioResources, Via P. Castellino 111, 80131 Naples, Italy;

[4]*National Gene Bank of Tunisia (BNG), Tunis, Tunisia;*

[5]*Agricultural Applied Biotechnology Laboratory (LR16INRAT06), Institut National de la Recherche Agronomique de Tunisie (INRAT), University of Carthage, Tunis, Tunisia.*

[6]Institute of Biosciences and BioResources, Via G. Amendola 165/A, Bari, Italy

***Correspondence:**
Carmen Bianco, carmen.bianco@ibbr.cnr.it
Asma Guizani, guizani_asma9@yahoo.fr

1 Supplementary Tables

Table S1. P-values obtained from two-way analyses of variance (ANOVA) carried out for all studied parameters.

Parameters	P value Treatment	P value Genotype	P value G*T interaction
Ψ_π^{100}	$p < 0.001$	$p < 0.005$	$p > 0.05$
Ψ_π^{0}	$p < 0.001$	$p < 0.05$	$p < 0.05$
RWC_0	$p > 0.05$	$p > 0.05$	$p > 0.05$
AWC	$p < 0.001$	$p < 0.01$	$p < 0.001$
Ɛmax	$p < 0.001$	$p < 0.001$	$p < 0.001$
Proline	$p < 0.001$	$p < 0.001$	$p < 0.001$
Sucres	$p < 0.001$	$p < 0.001$	$p < 0.001$
Na^+	$p > 0.05$	$p > 0.05$	$p > 0.05$
K^+	$p > 0.05$	$p < 0.001$	$p < 0.05$
Ca^{2+}	$p > 0.05$	$p < 0.001$	$p > 0.05$
Ci	$p < 0.01$	$p > 0.05$	$p > 0.05$
E	$p < 0.05$	$p < 0.001$	$p < 0.001$
gs	$p < 0.01$	$p < 0.001$	$p < 0.005$
A	$p < 0.001$	$p < 0.001$	$p < 0.001$
WUE	$p < 0.001$	$p < 0.001$	$p < 0.001$
iWUE	$p < 0.01$	$p > 0.05$	$p > 0.05$
LA	$p < 0.001$	$p < 0.001$	$p < 0.005$
Chl	$p < 0.001$	$p > 0.05$	$p < 0.05$
$SD_{(lower)}$	$p < 0.001$	$p < 0.001$	$p < 0.001$
$SD_{(upper)}$	$p < 0.001$	$p < 0.05$	$p < 0.001$
MDA	$p < 0.001$	$p < 0.001$	$p < 0.001$
EL	$p < 0.001$	$p < 0.001$	$p < 0.001$
BY	$p < 0.001$	$p < 0.001$	$p < 0.001$
GY	$p < 0.001$	$p < 0.001$	$p < 0.05$
GN	$p < 0.001$	$p < 0.001$	$p < 0.05$
TGW	$p < 0.001$	$p < 0.001$	$p < 0.01$
AAO	$p < 0.001$	$p < 0.001$	$p < 0.001$
PIP2:1	$p < 0.001$	$p < 0.001$	$p < 0.001$
